# Modeling Influencing Factors in B-Cell Reconstitution After Hematopoietic Stem Cell Transplantation in Children

**DOI:** 10.3389/fimmu.2021.684147

**Published:** 2021-05-07

**Authors:** Nicolaas G. van der Maas, Erik G. J. von Asmuth, Dagmar Berghuis, Pauline A. van Schouwenburg, Hein Putter, Mirjam van der Burg, Arjan C. Lankester

**Affiliations:** ^1^ Willem-Alexander Children’s Hospital, Department of Pediatrics and Laboratory for Pediatric Immunology, Leiden University Medical Center, Leiden, Netherlands; ^2^ Leiden University Medical Center, Department of Medical Statistics and Bioinformatics, Leiden, Netherlands

**Keywords:** B lymphocyte, allogeneic, hematopoietic stem cell transplantation, immune reconstitution, pediatric, linear mixed effect modelling

## Abstract

Reduced total and memory B-cell numbers in peripheral blood long term after hematopoietic stem cell transplantation (HSCT) are associated with an increased incidence of infections and immune complications. Using novel modelling strategies, baseline factors influencing B-cell reconstitution can be comprehensively studied. This study aims to investigate the numerical total and memory B-cell reconstitution in children and the association with baseline determinants 0.5-2 years after allogeneic HSCT. Eligible for inclusion were children transplanted in our center between 2004-2017 who received a first HSCT for malignant or non-malignant disorders. The continuous absolute counts of total and memory B-cells were evaluated as outcome measure. Exploratory analysis at one year was done to identify possible determinants. Linear mixed effect modelling was used to analyze the association of these determinants with total and memory B-cell reconstitution 0.5-2 years after HSCT. In a cohort of 223 evaluable patients analyzed at 1-year after HSCT donor age, stem cell source, donor type, recipient age and conditioning were identified as significant determinants for total and memory B-cell numbers. Multivariable analysis revealed that both donor and recipient age were inversely correlated with the size of total and memory B-cell reconstitution. In contrast, no correlation was found with stem cell source, donor type and conditioning. Making use of linear mixed modelling both stem cell donor and recipient age were identified as independent determinants of total and memory B-cell reconstitution 0.5-2 years after HSCT.

## Highlights

- Using a novel linear mixed model approach we demonstrated that B-cell and memory B-cell numbers 0.5-2 years after HSCT are influenced by time after transplantation, donor age and recipient age.
- Stem cell source, donor type and conditioning do not influence the B-cell and memory B-cell numbers 0.5-2 years after HSCT.

## Introduction

Allogeneic hematopoietic stem cell transplantation (HSCT) is a curative treatment for patients with congenital and acquired disorders of the hematopoietic system. After HSCT, balanced recovery of the repopulating hematopoietic system is required to ensure lasting protective immunity and immune tolerance (1).

Typically, innate immune reconstitution precedes the recovery of the adaptive immune system. Full reconstitution of the hematopoietic system, especially B-cell immunity, can take up to 2 years or even longer. Immune reconstitution after allogeneic HSCT has been studied extensively with the main focus on T-cell reconstitution. Only limited information is available about B-cell reconstitution.

Following HSCT, proper B-cell reconstitution is required to provide optimal protective immunity against pathogens as well as balanced immune regulation. Deficiencies therein may result in lasting immunoglobulin (Ig) dependency, increased risk of infections, impaired vaccine responses and immune dysregulation, leading to considerable morbidity and mortality (2–4). Identifying baseline factors that influence the reconstitution pattern of peripheral B-cells could be instrumental to predict clinical and immunological transplant outcome, and to timely anticipate complications. So far, several transplant factors have been described to influence the numerical B-cell reconstitution in children including stem cell source, donor type and conditioning regimen [reviewed in (5)]. The impact of donor and recipient age as independent parameters on B-cell or memory B-cell reconstitution in pediatric HSCT recipients is largely unresolved. Still, evidence from the normal pediatric population shows dynamic age-dependent changes of the B-cell and memory B-cell populations during childhood (6).

Up to now, identification of determinants of B-cell and memory B-cell reconstitution after HSCT has been mainly performed with a suboptimal approach using single time point outcomes. This could potentially lead to data loss, as other time points are excluded, and precludes to study the influence of determinants on the pattern of reconstitution. Moreover, investigating the trajectories with multiple measurements per subject would provide a better view of the reconstitution kinetics after HSCT. Mixed effects models are designed to meet this requirement (7). As a first step, we studied in, to our knowledge, the largest pediatric cohort to date the numerical B-cell and memory B-cell reconstitution during the first half to two years after pediatric stem cell transplantation and their association with baseline factors using these mixed effects models after allogeneic HSCT.

## Methods

### Study Subjects

Eligible for inclusion were patients transplanted between April 2004 and January 2017 at the Leiden University Medical Center (LUMC). Inclusion criteria were children whom received a single bone marrow (BM), peripheral blood stem cell (PBSC) or cord blood (CB) HSCT from a matched family/sibling donor, haplo-identical donor or a matched/mismatched unrelated donor. Indication for transplantation was malignant hematological disease, non-malignant hematological disorders, and inborn errors of immunity. Because we wanted to investigate the full reconstitution pattern of B-cell and memory B-cell numbers after HSCT, we tried to exclude all possible complicated trajectories: death or relapse within half a year post-HSCT, use of rituximab and graft-versus-host disease more than grade II. Peripheral blood samples were routinely obtained for analysis at several time points after HSCT. All available blood samples between 0.5 and 2 years were used in the analysis. Missing outcome values were excluded from analysis. Transplantations were performed in line with the guidelines of the European society of Blood and Marrow Transplantation. Approval for obtaining and analyzing blood samples was given by the local ethics committee (protocol P01.028). Informed consent was provided by the patient and/or parent or guardian.

### Flow Cytometry

Between October 1, 2004 and December 31, 2015, PBMC were separated using ficoll-isopaque density gradient centrifugation. PBMC were stained with CD45, CD14, CD33, CD235a, CD19, CD27 antibodies. Four-color flow cytometry was performed on a BD FACS Calibur II flow cytometer (Becton Dickinson Biosciences [BD], Franklin Lakes, NJ). Between January 1, 2016 and January 1, 2019, leukocyte subsets (absolute leukocyte counts and differential) were measured by an automated hematology analyzer, and lymphocyte subpopulations (CD3+ T cells, CD3+CD4+ T cells, CD3+CD8+ T cells, CD19+ B cells and CD16+/-CD56+ NK cells, CD19+CD27+ memory B-cells) were measured by flow cytometry in freshly collected blood samples as part of the patients’ routine clinical follow-up. Data were analyzed using BD Cellquest software. Lymphocytes were defined as CD45+ CD33/CD235a/CD14− cells within the forward/sideward scatter lymphocyte gate. Total B-cells were defined as CD19+ cells and total memory B-cells as CD19+CD27+ cells within the lymphocyte gate and absolute cell numbers per μL of peripheral blood were calculated.

### Outcome

The outcome was the continuous count of total and memory B-cells.

### Statistical Analysis

To assess what potential factors are associated with higher or lower B-cell counts we started with determinant analysis at 1 year. Median and interquartile ranges were used to describe continuous variables. Donor and recipient age were categorized in 0-5, 5-10, 10-15 and >15 years. The variables were compared on total and memory B-cell count using the Wilcoxon signed-rank test or Kruskal-Wallis test. We constructed a regression model in which the variables from analysis at 1 year were selected. To assess the trajectories with multiple measurements per patient, Mixed effects models were used to investigate the patterns of total and memory B-cell count after HSCT. The total and memory B-cell count were the outcomes of the model. To satisfy the model assumptions, the total B-cell and memory B-cell count was transformed with the natural logarithm. As independent variable, the time after HSCT was included. Time was allowed to have a non-linear effect, using a natural cubic spline. Adding splines in a model enables flexibility, capturing the trend in the data with more precision. The spline knots were set on 9 and 13 months. Residual plots were used to validate the models’ assumptions.

The statistical analyses were performed with R software version 3.6.2 using the nlme (linear mixed effects model) software package (8, 9).

## Results

### Included Patients

Between April 2004 and January 2017, 359 patients received a first HSCT at the LUMC (**Figure S1**). First, 68 patients were excluded (19%) because of death or relapse of disease within 6 months after HSCT. Subsequently, 44 patients (12%) were excluded from the cohort because of the use of rituximab, a B-cell depleting monoclonal antibody. Acute graft versus host disease (aGvHD) above grade 2 was present in 21 patients (6%). These patients were also excluded, because of the intensive and prolonged immunosuppressive treatment associated with aGvHD. Finally, 3 patients (0.8%) were excluded who had no engraftment or from whom no blood samples were available in the period of analysis. This resulted in a sample population of 223 patients (**Figure S1**). From these patients, we obtained 1531 data points for total B-cell numbers of all 223 patients and 993 data points for memory B-cell numbers of 219 patients. The mean follow up time of the cohort was 1.7 years with a median of 6 measurements per patient for total B-cell numbers. For memory B-cell numbers, the mean follow up time was 1.4 years with a median of 4 measurements per patient.

### Patient Characteristics

Patient characteristics are summarized in **Table 1**. The subjects in the sample population were predominantly male (69.5%) with a median age of 8.7 years. The median donor age (CB excluded in this analysis) was 24.6 years and there was a balanced use of male and female donors in the group (male 50.2% vs female 49.8%). Most patients were transplanted with a bone marrow graft (78.5%), and had an unrelated donor (53.8%). Indication for transplantation was in most cases malignant disease (48.9%). In 207 (92.8%) patients myeloablative conditioning (MAC) was used and in 16 (7.2%) reduced intensity conditioning (RIC).

**Table 1:** Patient characteristics.

** **		**Total = 223**
Recipient age	Year	8.7 (0.2 -17.9)
Recipient sex	Male	155 (69.5%)
	Female	68 (30.5%)
Donor age (CB excluded)	Year	24.6 (1.75-56.8)
Donor sex	Male	112 (50.2%)
	Female	111 (49.8%)
Stem cell source	BM	175 (78.5%)
	CB	22 (9.9%)
	PBSC	26 (11.7%)
Donor type	Identical related	83 (37.2%)
	Haplo-identical	20 (9.0%)
	Unrelated	120 (53.8%)
HSCT indication	Malignant hematological disease	109 (48.9%)
	Non-malignant hematological disorders	82 (36.8%)
	Inborn errors of immunity	32 (14.3%)
Conditioning	MAC	207 (92.8%)
	RIC	16 (7.2%)

BM, bone marrow; CB, cord blood; PBSC, peripheral blood stem cells; MAC, myeloablative; RIC, reduced intensity conditioning. Data is presented as number with percentage (%) or as median with minimum and maximum range.

### Determinant Analysis at 1 Year

**Figure 1:**
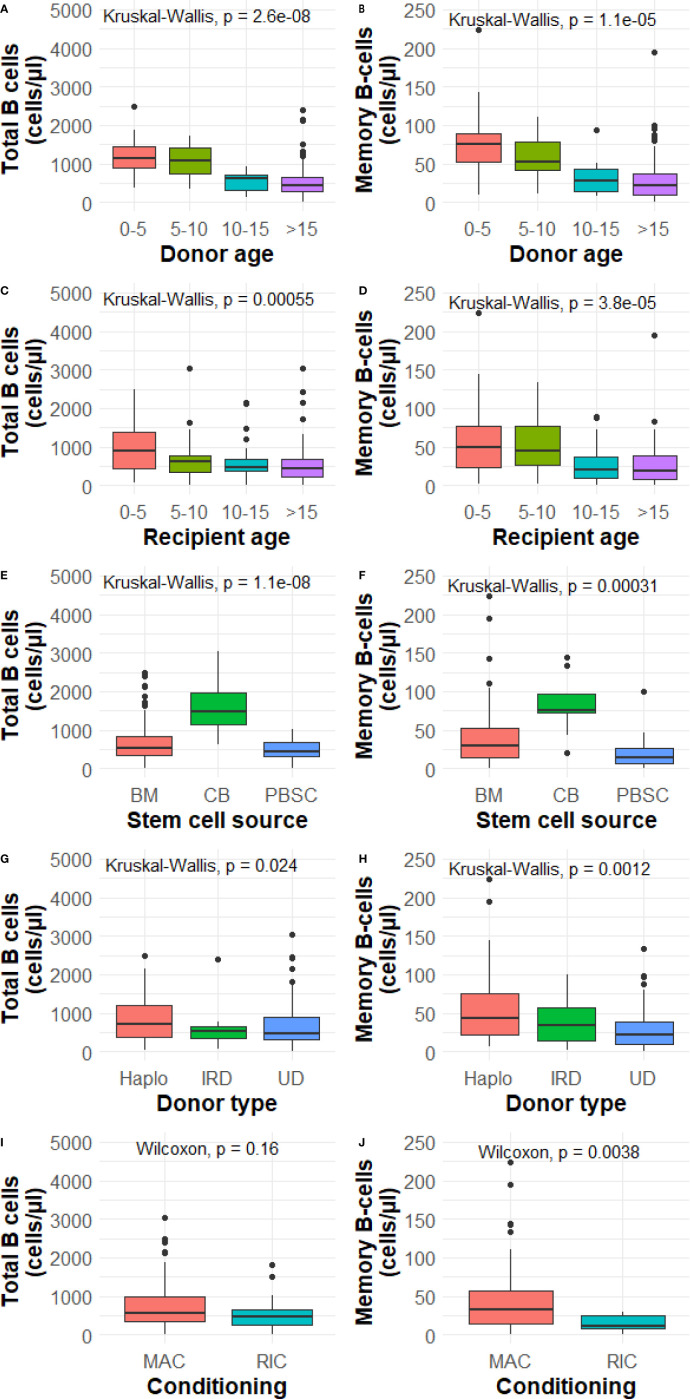
**(A–J)** Determinant analysis at 1 year. BM, bone marrow; CB, cord blood; PBSC, peripheral blood stem cells; Haplo, haplo-identical; IRD, identical related donor; UD, unrelated donor; MAC, myeloablative; RIC, reduced intensity conditioning. P value from the Wilcoxon signed-rank test or Kruskal-Wallis test comparing categories and total and memory B-cell numbers. Data is presented as median with interquartile ranges.

We investigated donor age (CB excluded), recipient age, stem cell source, donor type and conditioning as potential influencing factors on total and memory B-cell numbers at 1 year after HSCT. With the exception of conditioning and B-cells, all investigated determinants were significantly associated with total B and memory B-cell numbers 1 year after HSCT (**Figures 1A–J**). Subsequently, we investigated the determinants further in a multivariable analysis.

### B-Cell Reconstitution

**Figure 2:**
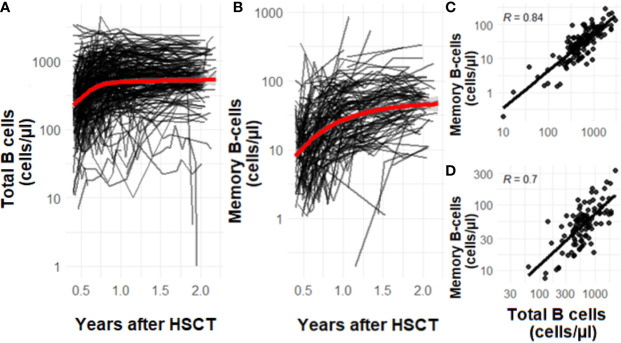
**(A–D)** The effect of time on total **(A)** and memory B-cell **(B)** numbers after HSCT. The correlation of memory B-cells and total B-cells 1 year (**C**, n=223) after HSCT and 2 years (**D**, n=86) after HSCT. Data is presented as individual trajectories and dots in black and as the average reconstitution in red. R is the Pearson’s correlation coefficient.

To study the dynamics of total and memory B-cell patterns over time in our sample population, we quantified cell numbers of all patients between 0.5-2 years after transplantation. Total and memory B-cell numbers increase over time after HSCT. The average total B-cell numbers increase in the first year after HSCT (**Figure 2A**). From 1 year after HSCT onwards, the B-cell numbers stabilize. The average memory B-cell numbers show a different profile with a continuous declining increase over two years (**Figure 2B**). Both for total as well as memory B-cell numbers considerable variability is seen up to 2 years after HSCT (**Figures 2A, B**). The total and memory B-cell numbers are correlated at 1 year and 2 years after HSCT (1 year after HSCT R=0.84 and 2 years after HSCT R=0.7, **Figures 2C, D**)

### Linear Mixed Effects Modelling

**Figure 3:**
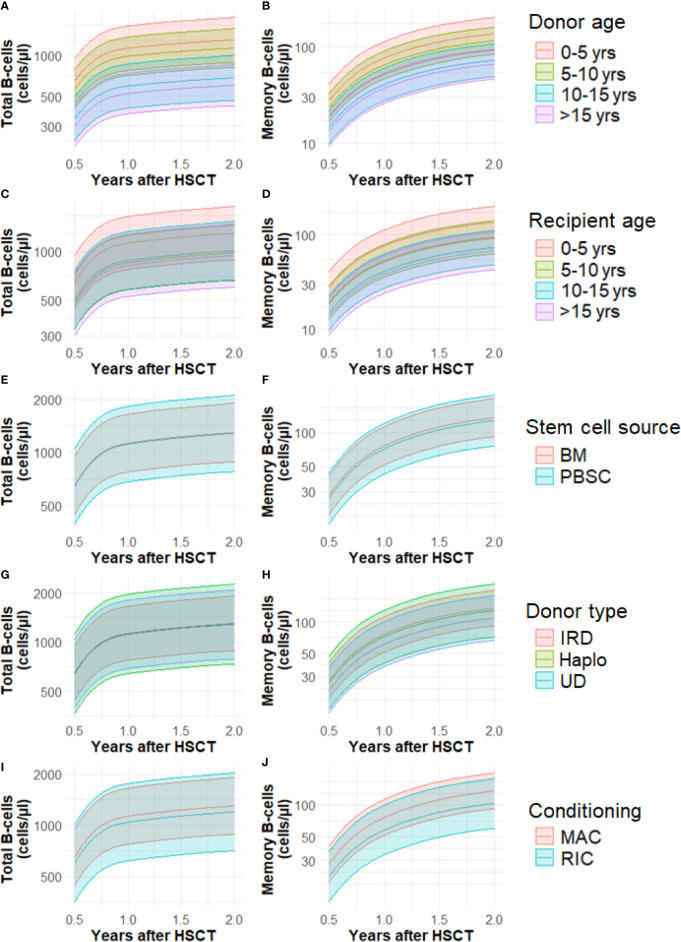
**(A–J)** Cell number trajectories of total B-cells and memory B-cells and the effect of baseline factors over time. Yrs, years; BM, bone marrow; CB, cord blood; PBSC, peripheral blood stem cells; Haplo, haplo-identical; IRD, identical related donor; UD, unrelated donor; MAC, myeloablative; RIC, reduced intensity conditioning. Lines represent mean predicted values, shaded areas the 95% CI’s.

We developed a model with time, donor age, recipient age, stem cell source, donor type and conditioning as independent variables. CB was left out of the analysis. Effect estimates can be seen in **Table S1** and in **Figures 3A–J** we plotted the effect of donor age, recipient age, stem cell source, donor type and conditioning for cell number trajectories over time.

#### Time After HSCT

Time after HSCT (0.5-2 years) has a significant influence on the reconstitution of total and memory B-cell numbers. Total B-cells increase significantly between 6-9 months (P< 0.001, **Table S1**), 9-13 months (P< 0.001) and 13-24 months (P< 0.001). Memory B-cell numbers show a similar trend, they increase significantly between 6-9 months (P< 0.001), 9-13 months (P< 0.001) and 13-24 months (P< 0.001).

#### Donor Age

Patients transplanted with younger donors have a significantly increased total and memory B-cell trajectories at 6 months. This difference remains visible up to 2 years after HSCT (**Figures 3A, B**). For total B-cells, we found no significant difference between the trajectories of patients receiving a graft of 0-5 years old donor and 5-10 years (P=0.56, **Table S1**, **Figure 3A**). However, the adjusted mean trajectory was decreased for those receiving grafts with donor age 10-15 years and also for those receiving grafts with donor age >15 years compared to donor age 0-5 years (P=0.01, P=0.001). The trajectories of memory B-cells in patients receiving a graft of 0-5 years and 5-10 years old donor were not significantly different (P=0.51, **Figure 3B**). The adjusted mean trajectory was less for those receiving grafts with donor age 10-15 years and donor age >15 years compared to donor age 0-5 years (P=0.009, P=0.004). The linear association of total and memory B-cells and donor age can be seen in **Figures S2 A, B**.

#### Recipient Age

Recipient age 5-10 is associated with a lower total B-cell number mean adjusted trajectory 0.5-2 years after HSCT compared to the reference recipient age of 0-5 years (P=0.04, **Table S1**, **Figure 3C**). Recipient age 10-15 was not significantly different (P=0.07), but patient older than 15 years again had lower total B-cell numbers (P=0.04). Patient age 5-10 years did not significantly influence memory B-cell numbers (P=0.11, **Figure 3D**). But patients between 10-15 years and older than 15 years show lower memory B-cell numbers after HSCT compared to reference (P<0.001). The linear association of total and memory B-cells and recipient age can be seen in **Figure S3 A, B**.

#### Stem Cell Source, Donor Type and Conditioning

There were no significant variations in the trajectories with respect to the total and memory B-cell numbers and the use of different stem cell sources (BM, PBSC), donor type (identical related, haplo-identical or unrelated donor) and conditioning used prior to HSCT (MAC or RIC) (**Table S1**, **Figures 3E–J**).

## Discussion

This study aimed to identify baseline characteristics that influence the total and memory B-cell reconstitution after HSCT in children. We used a comprehensive analysis approach, taking into account the grouped nature of our data with linear mixed modelling. Making optimal use of all available data from each patient, we found an inverse correlation with donor age and recipient age. Stem cell source, donor type and conditioning revealed no significant association with total and memory B-cell reconstitution trajectories in multivariable analysis.

Our data show that donor age has an inverse relationship with total and memory B-cell reconstitution. The effect of donor age on the total and memory B-cell reconstitution could be a reflection of physiological age-associated quantitative changes. Age dependent dynamics are observed in all B-cell subsets of the normal pediatric (donor)population (6) and could determine the pattern of immune reconstitution together with recipient age, time after HSCT and possibly environmental factors. In line with these findings, it will be interesting to address the potential association between donor age and infection susceptibility or protective vaccination responses after HSCT in future studies. Although several other studies have analyzed the effect of donor age on B-cell reconstitution, our study is the first to use the mixed model approach to investigate the influence of donor age on the total and memory B-cell reconstitution after HSCT (10–12). Avanzini et al. and Gonzalez-Vicent et al. studied the percentage of B-cells instead of absolute B-cell numbers, and found no association with donor age (10, 11). Storek et al. reported a correlation between peripheral B cell counts and the number of B cell precursors as detected in bone marrow during the first year after HSCT (12). However the latter were not related to the numbers of CD34 cells in the graft, the stem cell source, and donor and recipient age. Together, this suggests that both age-related donor cell-intrinsic factors and quantitative changes in composition of the donor graft may have an impact on B cell reconstitution. As younger donors appear to be associated with improved B-cell reconstitution, this aspect may be taken into consideration during the process of donor selection.

We found that younger recipient age was associated with higher total and memory B-cell counts after HSCT pointing to a potential role of non-hematopoietic/microenvironmental factors. In mice studies, transplanted young hematopoietic stem cells engraft at a lower efficiency in aged recipients, give rise to a lower percentage of donor derived B-cells and seem to adversely impact mature B-cell production short term, but not long term after HSCT (13, 14). Transplanting hematopoietic stem cells into a younger microenvironment might thus contribute to a numerical increase of the B-cell reconstitution as observed in our study. Further studies will be required to unravel the age-related mechanisms responsible for the hematopoietic cell-intrinsic as well as microenvironmental determinants of B cell development.

RIC regimens are frequently used in children either because the underlying disease or the pre-existing co-morbidities prohibits a MAC approach. Conditioning with RIC is associated with better survival in these subgroups, due to favorable toxicity profile and thus lower transplant-related mortality (15, 16). Since RIC results in less myeloablation it is more likely for autologous hematopoietic precursors including B-cell lineage, to survive. In this study we did not have the opportunity to investigate whether B-cell chimerism had an impact on numerical B-cell reconstitution. Still, in our study RIC and MAC had similar B-cell and memory B-cell reconstitution profiles.

This study demonstrates that there is no significant difference in B-cell reconstitution beyond 6 months after HSCT between BM and PBSC as stem cell source, which is in line with previous reports (17, 18). In contrast, CB has a significantly higher B-cell and memory B-cell numbers 1 year after HSCT, which is in line with other studies (2, 19). The difference could be explained by the higher number of B lymphocyte progenitors found in CB with a better *in vitro* and *in vivo* B-cell reconstituting capacity compared to bone marrow grafts (20). Our study shows that there was no significant difference between identical related, unrelated or haplo-identical donors with respect to numerical B-cell or memory B-cell reconstitution. This observation could be relevant in the context of donor selection and the increasing use of haploidentical donors.

In this retrospective study in which we focused on the quantitative reconstitution, memory B-cells were defined based on positivity of the classical markers CD19 and CD27. The CD19+CD27+ memory B-cell population includes both non-switched and switched memory B-cells. Furthermore, CD27- memory B-cells, accounting for approximately 2.5-8.2% of all B-cells in the healthy pediatric population, are not included here (21). This study was a single center analysis. In the future, the general concept of model-based evaluation of clinical parameters should preferably be implemented in prospective studies. Detailed data on the other B-cell subsets (transitional, naïve, non-switched and switched), the B-cell receptor repertoire (both naïve and antigen selected) and the quality of humoral immune response e.g. vaccine responses or infection rate after HSCT gathered through multicenter prospective cohort studies will increase our understanding of the fitness of the reconstituting adaptive immune system. More specifically, this could validate the findings in this study, gain new insights in successful adaptive immune reconstitution and stimulate the integration of model-informed evaluation in daily clinical practice with the aim to optimize clinical and immunological outcome after HSCT.

In conclusion, using a linear mixed modelling approach we demonstrated in a large pediatric cohort that donor age, recipient age as well as time after HSCT have a significant impact on the reconstitution pattern of total and memory B-cell numbers 0.5-2 years after HSCT.

## Data Availability Statement

The data and code of the model is available on request to the corresponding author.

## Ethics Statement

The studies involving human participants were reviewed and approved by Medisch Ethisch Toetsingscommissie Leiden-Den Haag-Delft, Leiden University Medical Center. Written informed consent to participate in this study was provided by the participants’ legal guardian/next of kin.

## Author Contributions

NM and MB contributed conception of the paper. NM did the statistical analysis and wrote the first draft of the manuscript. HP and EA contributed to the statistical analysis. DB, MB, PS, and AL contributed to study design and contributed in writing the manuscript. All authors contributed to the article and approved the submitted version.

## Conflict of Interest

The authors declare that the research was conducted in the absence of any commercial or financial relationships that could be construed as a potential conflict of interest.

## References

[1] Ogonek J Kralj Juric M Ghimire S Varanasi PR Holler E Greinix H. Immune Reconstitution After Allogeneic Hematopoietic Stem Cell Transplantation. Front Immunol (2016) 7:507. 10.3389/fimmu.2016.00507 27909435 PMC5112259

[2] Abdel-Azim H Elshoury A Mahadeo KM Parkman R Kapoor N. Humoral Immune Reconstitution Kinetics After Allogeneic Hematopoietic Stem Cell Transplantation in Children: A Maturation Block of IgM Memory B Cells May Lead to Impaired Antibody Immune Reconstitution. Biol Blood Marrow Transpl (2017) 23:1437–46. 10.1016/j.bbmt.2017.05.005 28495643

[3] Corre E Carmagnat M Busson M de Latour RP Robin M Ribaud P. Long-Term Immune Deficiency After Allogeneic Stem Cell Transplantation: B-cell Deficiency is Associated With Late Infections. Haematologica (2010) 95:1025–9. 10.3324/haematol.2009.018853 PMC2878804 20133894

[4] Sarantopoulos S Blazar BR Cutler C Ritz J. B Cells in Chronic Graft-Versus-Host Disease. Biol Blood Marrow Transplant (2015) 21:16–23. 10.1016/j.bbmt.2014.10.029 25452031 PMC4295503

[5] van der Maas NG Berghuis D van der Burg M Lankester AC. B Cell Reconstitution and Influencing Factors After Hematopoietic Stem Cell Transplantation in Children. Front Immunol (2019) 10:782. 10.3389/fimmu.2019.00782 31031769 PMC6473193

[6] Blanco E Perez-Andres M Arriba-Mendez S Contreras-Sanfeliciano T Criado I Pelak O. Age-Associated Distribution of Normal B-cell and Plasma Cell Subsets in Peripheral Blood. J Allergy Clin Immunol (2018) 141:2208–19.e2216. 10.1016/j.jaci.2018.02.017 29505809

[7] von Asmuth EGJ Mohseny AB Putter H Schilham MW Lankester AC. Modeling Long-Term Erythropoietic Recovery After Allogeneic Stem Cell Transplants in Pediatric Patients. Front Pediatr (2020) 8:584156. 10.3389/fped.2020.584156 33330281 PMC7734089

[8] R Core Team. R: A Language and Environment for Statistical Computing: R Foundation for Statistical Computing. (2019). Available at: http://www.R-project.org/.

[9] Pinheiro J BD DebRoy S Sarkar D Core Team R. Nlme: Linear and Nonlinear Mixed Effects Models. R Package Version, Vol. 3. CRAN (2018). pp. 1–137.

[10] Avanzini MA Locatelli F Dos Santos C Maccario R Lenta E Oliveri M. B Lymphocyte Reconstitution After Hematopoietic Stem Cell Transplantation: Functional Immaturity and Slow Recovery of Memory CD27+ B Cells. Exp Hematol (2005) 33:480–6. 10.1016/j.exphem.2005.01.005 15781339

[11] Gonzalez-Vicent M Molina B Deltoro N Sevilla J Vicario JL Castillo A. Donor Age Matters in T-cell Depleted Haploidentical Hematopoietic Stem Cell Transplantation in Pediatric Patients: Faster Immune Reconstitution Using Younger Donors. Leuk Res (2017) 57:60–4. 10.1016/j.leukres.2017.03.001 28292719

[12] Storek J Wells D Dawson MA Storer B Maloney DG. Factors Influencing B Lymphopoiesis After Allogeneic Hematopoietic Cell Transplantation. Blood (2001) 98:489–91. 10.1182/blood.v98.2.489 11435323

[13] Rossi DJ Bryder D Zahn JM Ahlenius H Sonu R Wagers AJ. Cell Intrinsic Alterations Underlie Hematopoietic Stem Cell Aging. Proc Natl Acad Sci USA (2005) 102:9194–9. 10.1073/pnas.0503280102 PMC1153718 15967997

[14] Ergen AV Boles NC Goodell MA. Rantes/Ccl5 Influences Hematopoietic Stem Cell Subtypes and Causes Myeloid Skewing. Blood (2012) 119:2500–9. 10.1182/blood-2011-11-391730 PMC3311273 22289892

[15] Chiesa R Veys P. Reduced-Intensity Conditioning for Allogeneic Stem Cell Transplant in Primary Immune Deficiencies. Expert Rev Clin Immunol (2012) 8:255–66; quiz 267. 10.1586/eci.12.9 22390490

[16] Rao K Amrolia PJ Jones A Cale CM Naik P King D. Improved Survival After Unrelated Donor Bone Marrow Transplantation in Children With Primary Immunodeficiency Using a Reduced-Intensity Conditioning Regimen. Blood (2005) 105:879–85. 10.1182/blood-2004-03-0960 15367433

[17] Storek J Dawson MA Storer B Stevens-Ayers T Maloney DG Marr KA. Immune Reconstitution After Allogeneic Marrow Transplantation Compared With Blood Stem Cell Transplantation. Blood (2001) 97:3380–9. 10.1182/blood.v97.11.3380 11369627

[18] Abrahamsen IW Somme S Heldal D Egeland T Kvale D Tjonnfjord GE. Immune Reconstitution After Allogeneic Stem Cell Transplantation: The Impact of Stem Cell Source and Graft-Versus-Host Disease. Haematologica (2005) 90:86–93. Available at: https://www.haematologica.org/article/download/3348/12043. 15642674

[19] Bartelink IH Belitser SV Knibbe CAJ Danhof M de Pagter AJ Egberts TCG. Immune Reconstitution Kinetics as an Early Predictor for Mortality Using Various Hematopoietic Stem Cell Sources in Children. Biol Blood Marrow Transpl (2013) 19:305–13. 10.1016/j.bbmt.2012.10.010 23092812

[20] Arakawa-Hoyt J Dao MA Thiemann F Hao QL Ertl DC Weinberg KI. The Number and Generative Capacity of Human B Lymphocyte Progenitors, Measured *In Vitro* and *In Vivo*, is Higher in Umbilical Cord Blood Than in Adult or Pediatric Bone Marrow. Bone Marrow Transpl (1999) 24:1167–76. 10.1038/sj.bmt.1702048 10642804

[21] van Gent R van Tilburg CM Nibbelke EE Otto SA Gaiser JF Janssens-Korpela PL. Refined Characterization and Reference Values of the Pediatric T- and B-cell Compartments. Clin Immunol (2009) 133:95–107. 10.1016/j.clim.2009.05.020 19586803

