# Supplementary material for: Modeling Influencing Factors in B-Cell Reconstitution After Hematopoietic Stem Cell Transplantation in Children
Source: Front Immunol. 2021 May 7;12:684147. doi: 10.3389/fimmu.2021.684147 (PMC8138425; doi:10.3389/fimmu.2021.684147)
Supplement: Supplementary file 1 [file DataSheet_1.docx]

Supplementary Material

**
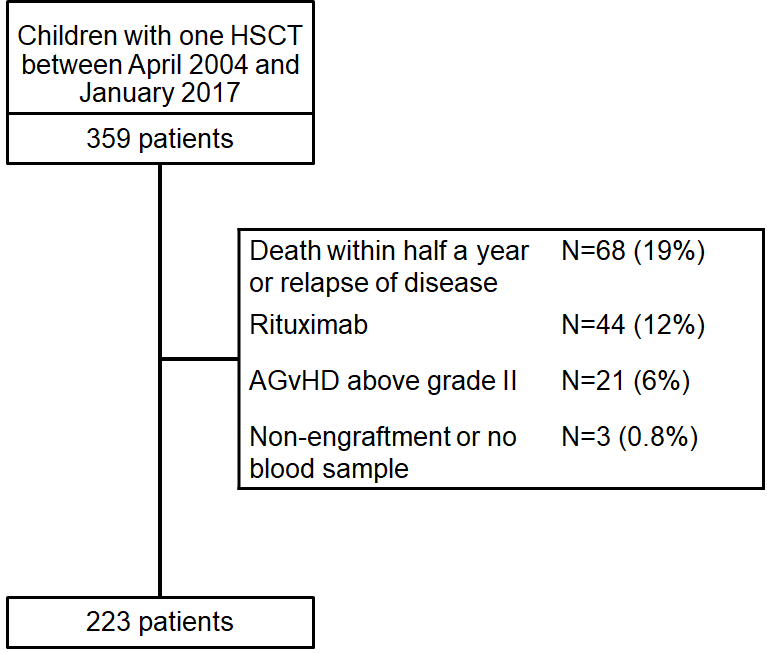
**

**Figure 1S.** Flowchart of patient selection, resulting in the sample population.


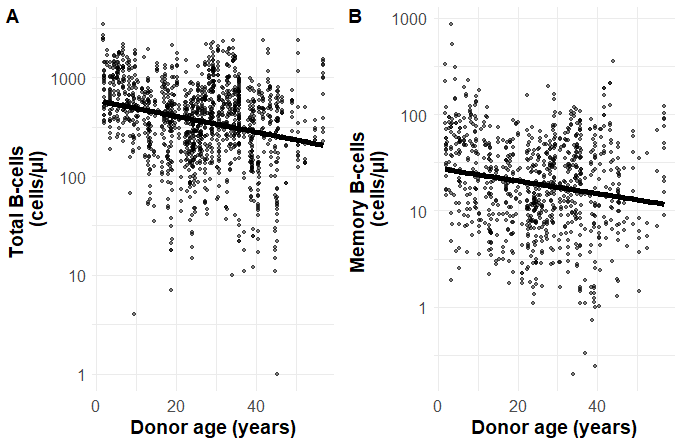


**Figure 2S A-B.** Cell numbers of total B-cells and memory B-cells and the effect of donor age. Lines represented observed mean values and dots the individual observations.


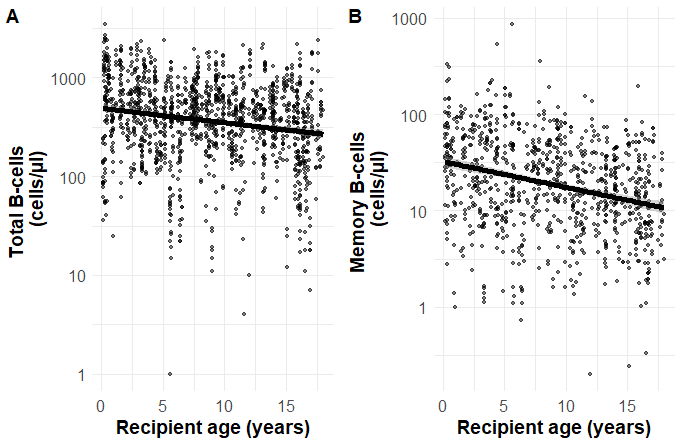


**Figure 3S A-B.** Cell numbers of total B-cells and memory B-cells and the effect of recipient age. Lines represented observed mean values and dots the individual observations.

**Table 1S.** Estimates of mixed effects model.

| **Model** | **Determinant** | **Significance** | **Effect estimate** | **95% CI** |
| --- | --- | --- | --- | --- |
| Total B-cells | Intercept | <0.001 | 6.24 | 5.85 – 6.62 |
|  | Time 6-9 months | <0.001 | 0.61 | 0.43 – 0.79 |
|  | Time 9-13 months | <0.001 | 1.53 | 1.30 – 1.76 |
|  | Time 13-24 months | <0.001 | 0.60 | 0.44 – 0.77 |
|  | Donor age 0-5 |  | 1(ref) |  |
|  | Donor age 5-10 | 0.56 | -0.14 | -0.60 – 0.33 |
|  | Donor age 10-15 | 0.01 | -0.65 | -1.13 – -0.16 |
|  | Donor age >15 | 0.001 | -0.77 | -1.24 – -0.30 |
|  | Recipient age 0-5 |  | 1 (ref) |  |
|  | Recipient age 5-10 | 0.04 | -0.28 | -0.54 – -0.02 |
|  | Recipient age 10-15 | 0.07 | -0.25 | -0.52 – 0.02 |
|  | Recipient age >15 | 0.04 | -0.32 | -0.62 – -0.02 |
|  | BM |  | 1(ref) |  |
|  | PBSC | 0.64 | -0.01 | -0.33 – 0.30 |
|  | Identical related |  | 1(ref) |  |
|  | Haplo-identical | 0.96 | -0.01 | -0.43 – 0.41 |
|  | Unrelated | 0.92 | -0.02 | -0.33 – 0.29 |
|  | MAC |  | 1(ref) |  |
|  | RIC | 0.67 | -0.08 | -0.45 – 0.29 |
| Memory B-cells | Intercept | <0.001 | 2.97 | 2.55 – 3.40 |
|  | Time 6-9 months | <0.001 | 1.41 | 1.19 – 1.64 |
|  | Time 9-13 months | <0.001 | 2.65 | 2.35 – 2.95 |
|  | Time 13-24 months | <0.001 | 1.48 | 1.29 – 1.66 |
|  | Donor age 0-5 |  | 1(ref) |  |
|  | Donor age 5-10 | 0.51 | -0.17 | -0.67 – 0.34 |
|  | Donor age 10-15 | 0.009 | -0.70 | -1.23 – -0.18 |
|  | Donor age >15 | 0.004 | -0.76 | -1.27 – -0.25 |
|  | Recipient age 0-5 |  | 1(ref) |  |
|  | Recipient age 5-10 | 0.11 | -0.24 | -0.52 – 0.05 |
|  | Recipient age 10-15 | <0.001 | -0.56 | -0.86 – -0.27 |
|  | Recipient age >15 | <0.001 | -0.63 | -0.97 – -0.30 |
|  | BM |  | 1(ref) |  |
|  | PBSC | 0.79 | -0.05 | -0.40 – 0.30 |
|  | Haplo-identical |  | 1(ref) |  |
|  | Identical related | 0.87 | -0.04 | -0.50 – 0.42 |
|  | Unrelated | 0.26 | -0.19 | -0.53 – 0.14 |
|  | MAC |  | 1(ref) |  |
|  | RIC | 0.31 | -0.21 | -0.63 – 0.20 |

BM = bone marrow, PBSC = peripheral blood stem cells, MAC = myeloablative. RIC = reduced intensity conditioning, 95% CI = 95% confidence interval.
